# Tuberculous Pericarditis in an Immunocompromised Patient: A Case Report

**DOI:** 10.7759/cureus.71507

**Published:** 2024-10-15

**Authors:** Nawaf Alotaibi, Faisal Almutawa, Alwaleed Alhazzaa, Ihab Suliman

**Affiliations:** 1 College of Medicine, King Saud Bin Abdulaziz University for Health Sciences, Riyadh, SAU; 2 Internal Medicine, King Saud Bin Abdulaziz University for Health Sciences, Riyadh, SAU; 3 Basic Medical Sciences, King Saud Bin Abdulaziz University for Health Sciences, Riyadh, SAU; 4 Cardiology, King Abdulaziz Medical City, King Abdulaziz Cardiac Center, Ministry of National Guard Health Affairs, Riyadh, SAU

**Keywords:** antituberculosis therapy, constrictive pericarditis, constrictive tuberculous pericarditis, pericardial effusion, pericardiectomy, rheumatoid arthritis, right-sided heart failure, tuberculosis

## Abstract

This case report details the presentation, diagnostic process, and management of tuberculous pericarditis (TBP) in a 43-year-old male with a history of chronic severe pericarditis, right-sided heart failure, and rheumatoid arthritis. The patient exhibited symptoms of chest pain and dyspnea and showed signs of ascites and mild lower limb edema. Our patient was found to have significant pericardial thickening and effusion, as well as necrotic lymph nodes. Diagnostic tests, including a positive QuantiFERON-TB test (QIAGEN, Hilden, Germany) and imaging studies, supported the diagnosis of TBP. Management involved a combination of anti-tuberculosis therapy and eventually, a radical pericardiectomy. This report underscores the importance of timely diagnosis and treatment in improving outcomes for patients with TBP.

## Introduction

Tuberculosis (TB) is one of the leading causes of death worldwide [1]. TB is caused by *Mycobacterium tuberculosis* and is mainly transmitted through air. Clinical manifestations of TB vary but typically include fever, night sweats, weight loss, malaise, cough, and shortness of breath; however, patients could also present with atypical symptoms, which could complicate diagnosing the disease early. Although the lungs are the main target of mycobacterium, around 15-20% of patients develop extrapulmonary tuberculosis (EPTB) [2].

Tuberculous pericarditis (TBP) is seen in 1-2% of TB infections [3]. TBP can present as either constrictive pericarditis, pericardial effusion, or a combination of both [4]. TBP is the leading cause of constrictive pericarditis worldwide [5]. Early detection and timely treatment are essential for improving outcomes. In this report, we present a case of TBP in a 43-year-old immunocompromised patient with a history of chronic pericarditis and right-sided heart failure.

## Case presentation

A 43-year-old male presented to the emergency department (ED) with chest pain and heaviness for the past six hours. The chest pain was pleuritic and associated with palpitations, ascites, and mild lower leg edema. He had a medical history of rheumatoid arthritis, for which he had been treated with methotrexate, and chronic constrictive pericarditis leading to right-sided heart failure. His vital signs included a blood pressure of 103/69 mmHg, heart rate of 87 beats/min, respiratory rate of 19 breaths/min, and oxygen saturation (SpO2) of 100%. On examination, the patient was stable, experiencing mild to moderate pain, and was afebrile. Auscultation of the respiratory system revealed no pathologic findings. Cardiovascular assessment showed normal heart sounds with no additional sounds. The abdomen was soft and lax, with mild epigastric tenderness and ascites present.

**Figure 1:**
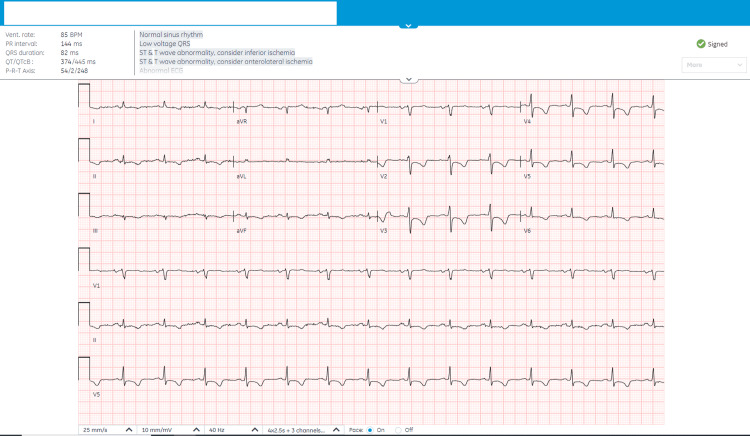
A 12-lead ECG recorded on 20/07/2023 demonstrated sinus rhythm with non-specific ST-T wave alterations and low voltage.

**Figure 2:**
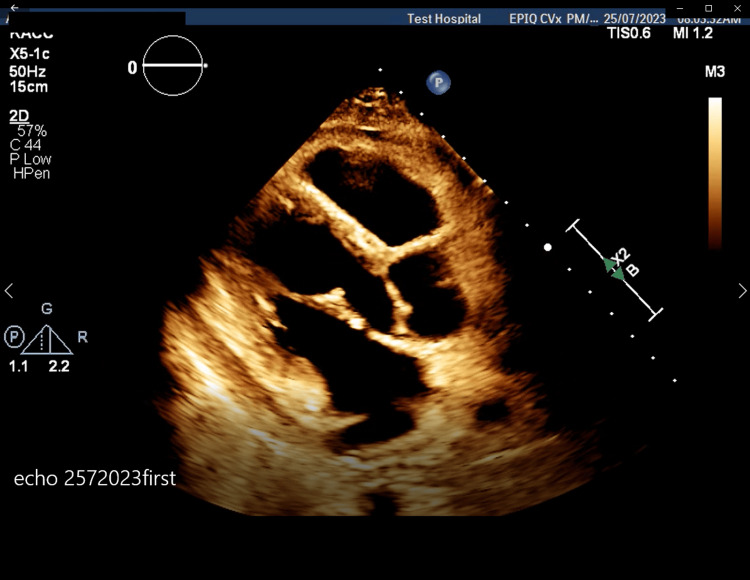
Echocardiography performed on 25/07/2023 revealed right ventricular dilation with mild to moderately reduced systolic function (RV free wall global longitudinal strain (RVFW-GLS): -15.9%), pericardial thickening, and a small pericardial effusion.

**Figure 3:**
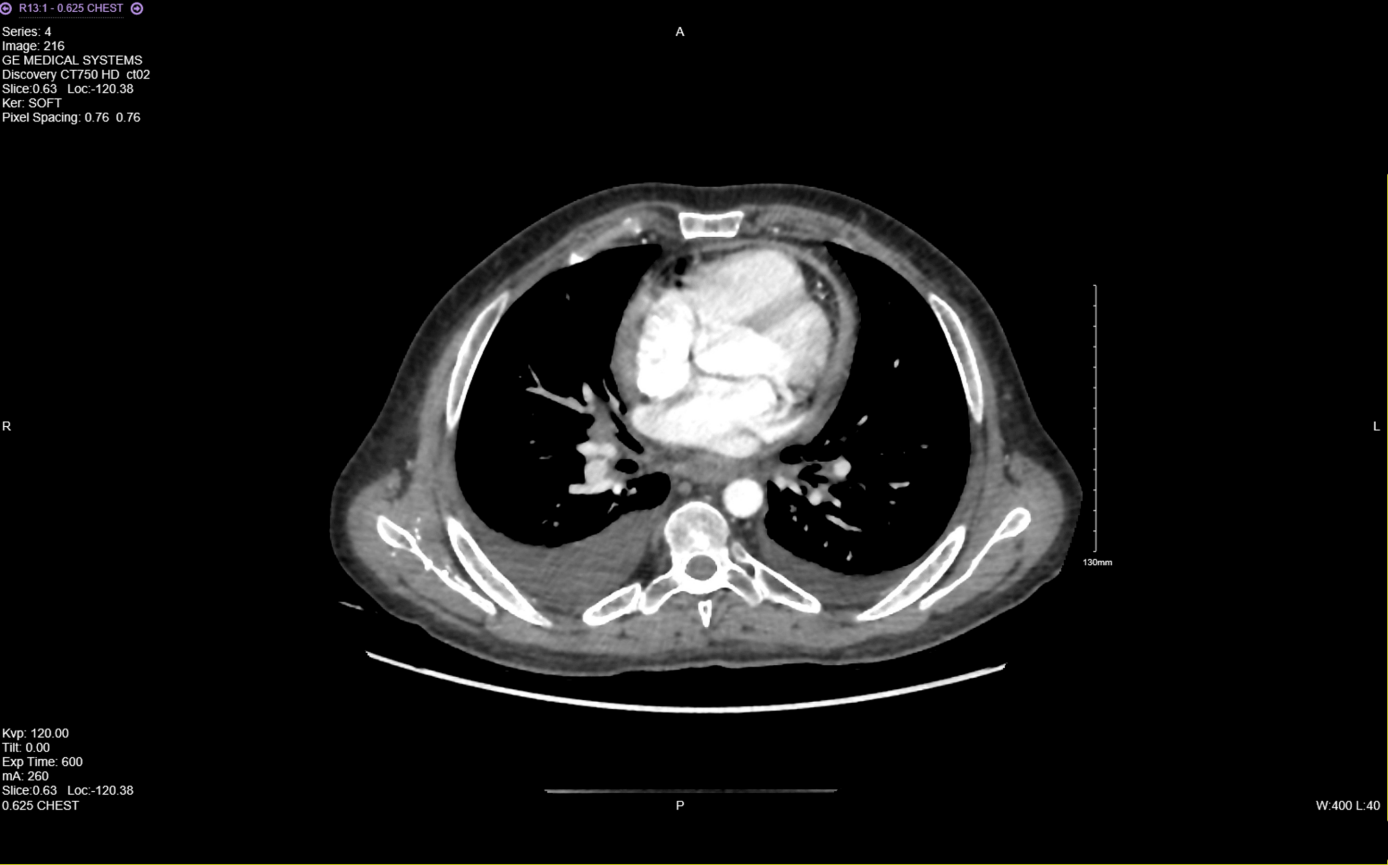
A CT scan conducted on 27/07/2023 showed bilateral mild to moderate pericardial effusion, along with significant pericardial thickening and enhancement.

In July 2023, his laboratory results at admission showed a brain natriuretic peptide (BNP) of 82.4 pmol/L, erythrocyte sedimentation rate (ESR) of 40 mm/hr, C-reactive protein (CRP) of 14 mg/L, and rheumatoid factor (RF) of 305 kU/L. A chest X-ray revealed minimal pleural effusion and an ECG (Figure 1) showed non-specific changes with normal sinus rhythm and low voltage. An echocardiogram (Figure 2) indicated right ventricle dilation with mild to moderately reduced systolic function (RV free wall global longitudinal strain (RVFW-GLS): -15.9%), pericardial thickening, and a small pericardial effusion. A CT scan (Figure 3) further detailed bilateral mild to moderate pericardial effusion with significant pericardial thickening and enhancement, multiple enlarged necrotic supraclavicular, mediastinal, and bilateral hilar lymph nodes, and mild bilateral pleural effusion. In August 2023, a QuantiFERON-TB test (QIAGEN, Hilden, Germany) returned positive, and right heart catheterization suggested constrictive pathology with a positive Kussmaul's sign and raised superior vena cava, right atrium, and right ventricular end-diastolic pressures. By October 2023, acid-fast bacillus (AFB) cultures showed no acid-fast bacilli. In November 2023, a biopsy of the supraclavicular lymph node was performed for TB, polymerase chain reaction (PCR), and AFB, and a left cervical lymph node biopsy revealed non-necrotizing granulomatous lymphadenitis with negative special stains for acid-fast bacilli and fungi; the PCR for *M. tuberculosis* complex was negative, and the biopsy was negative for malignancy. Despite negative AFB cultures and PCR results for *M. tuberculosis*, the clinical presentation and imaging findings were consistent with TBP.

**Figure 4:**
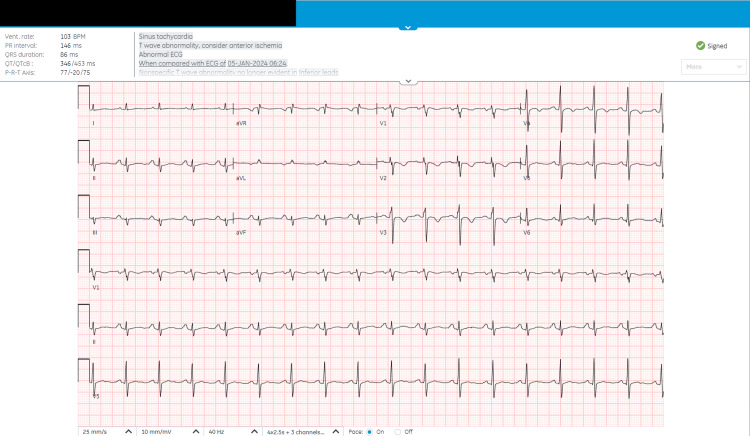
An ECG performed on 25/01/2024, post-pericardiectomy, demonstrated significant improvement, with non-specific T wave abnormalities no longer observed in the inferior leads.

Management included prednisone 10mg/day orally for rheumatoid arthritis that was treated previously with methotrexate 7.5mg/week but was discontinued due to his diagnosis of TBP. Furosemide 40mg/day per os for recurrent lower limb edema and ascites. TB treatment included rifampicin 600mg/day per os for 90 days, isoniazid 300mg/day per os for 90 days, pyrazinamide 40mg/day per os for 90 days, which was discontinued after 23 days due to pruritus, and ethambutol 1200mg/day per os for 90 days. Ultimately, in January 2024, a radical pericardiectomy was performed, and no further complications were noted afterward. Histopathological studies of the pericardium revealed chronic fibrosing pericarditis with necrotizing granulomas, confirming TB as the underlying cause. Upon follow-up, the patient was symptom-free, as well as ECG readings (Figure 4) showed significant improvements in which non-specific ST-T abnormalities were no longer evident in inferior leads. No additional symptoms or complications were reported.

## Discussion

We presented a 43-year-old male military officer with a history of chronic constrictive pericarditis, right-sided heart failure, and rheumatoid arthritis, which raised concerns about an underlying systemic illness. Given the patient's medical history and symptoms, there was a need to consider various differential diagnoses, including infectious, inflammatory, and neoplastic causes. TB seemed to be a potential cause due to the patient's immunocompromised history as he used methotrexate for two years for his rheumatoid arthritis. In addition, a positive QuantiFERON-TB test and chest CT findings of multiple enlarged and necrotic lymph nodes made TB the primary diagnosis.

If not treated effectively, TBP can result in severe complications, including cardiac tamponade, constrictive pericarditis, and even death. Studies show that 17-40% of patients die within six months of diagnosis [6]. TB is a major global health problem, and it can manifest as EPTB with a percentage of 20.9% of overall cases [7]. TBP can present as acute constrictive pericarditis, cardiac tamponade, or a combination of both. TBP is considered to be the leading cause of constrictive pericarditis worldwide and it is associated with a high mortality rate if not treated early [8].

The predominant symptoms of TBP are dyspnea, cough, and chest pain. Night sweats, weight loss, and edema are also common [9]. The most common signs are cardiomegaly, fever, and tachycardia [9]. Our patient had a variety of signs and symptoms including chest pain, dyspnea, night sweats, weight loss, and generalized edema. If low voltage is observed on the electrocardiogram with complexes measuring less than 5 mm in the limb leads and less than 10 mm in the precordial leads, large pericardial effusion should be suspected, as seen with our patient [10]. Chest X-ray allows visualization of pleural effusion; our patient had minimal pleural effusion on chest X-ray [4]. On the echocardiogram, TBP usually presents with pericardial effusion and thickening of the visceral pericardium [11]. This patient had a dilated right ventricle and systolic function was moderately reduced with pericardial thickening and small pericardial effusion. To help in assessing the volume and density of the fluid in the pericardium, as well as the thickness and enhancement of the pericardium, chest CT with contrast could be used [10]. Multiple enlarged necrotic supraclavicular, mediastinal, and bilateral hilar lymph nodes could be seen in patients with TBP [12].

Confirming a "definite" diagnosis of TBP requires the identification of tubercle bacilli in the fluid surrounding the pericardium or through microscopic examination of pericardial tissue samples. On the other hand, a "probable" diagnosis is made when there is evidence of TB elsewhere in the body in a patient with unexplained pericarditis, the presence of a lymphocytic pericardial fluid with elevated adenosine deaminase (ADA) levels, and/or a positive response to anti-TB treatment [13]. Typically, the pericardial fluid in TBP shows a protein-rich lymphocytic exudate that is frequently bloody. However, the fluid contains a low number of tubercle bacilli, and relying solely on smear examination yields an estimated diagnostic accuracy of only 5% [4]. The sensitivity of pericardial fluid culture ranges from 53% to 75%, but it takes approximately three weeks to obtain results [14]. PCR testing for *M. tuberculosis* DNA or RNA in pericardial fluid offers a more feasible and faster alternative with lower costs in comparison to PCR testing on pericardial tissue. However, its sensitivity is also lower (15% compared to 80%), and there is a risk of up to 20% false-positive results [15]. Pericardial ADA levels of ≥35 U/L are considered diagnostic for TBP, with a sensitivity of 90% and specificity of 74% [16].

A treatment regimen comprising rifampicin, isoniazid, pyrazinamide, and ethambutol for a minimum of two months, followed by isoniazid and rifampicin (totalling six months of therapy) has demonstrated great efficacy in effectively treating individuals with EPTB [17]. There is no significant improvement in outcomes with a treatment duration of nine months or more, and such extended regimens come with the drawbacks of higher expenses and lower patient adherence [17]. In addition, pericardiectomy is recommended for persistent constriction in the face of anti-TB medication but the timing of the surgery is controversial. There are varying recommendations among authors regarding the timing of pericardiectomy in relation to initiating chemotherapy. Some authors suggest performing pericardiectomy for all patients after starting chemotherapy [18], while others advocate reserving pericardiectomy for specific patients who do not respond to initial medical treatment [18]. A pericardiectomy is, therefore, recommended if the patient’s condition is static hemodynamically or deteriorates after four to eight weeks of anti-TB therapy [19]. If, however, the disease is associated with pericardial calcification, a marker of chronic disease, surgery should be undertaken earlier under anti-TB drug cover.

## Conclusions

This case report illustrates the importance of early recognition, accurate diagnosis, and prompt treatment of TBP in immunocompromised patients. The clinical history, symptoms, positive QuantiFERON-TB test, and chest CT findings supported the primary diagnosis of TBP. Timely recognition, accurate diagnosis, and prompt initiation of anti-TB therapy, including a regimen of rifampicin, isoniazid, pyrazinamide, and ethambutol, followed by isoniazid and rifampicin, are crucial for effective treatment. Pericardiectomy, if required, should be considered in cases of persistent constriction or deterioration after initial medical therapy. Understanding the complexities of TBP and its management is essential to improve outcomes and reduce morbidity and mortality associated with this serious manifestation of EPTB.
